# Meteorological Table

**Published:** 1813-01

**Authors:** 


### METEOROLOGICAL TABLE.

*From the 25th of November, to the 25th of December.*

| | D. | Therm. | | | Barom. | | Hygrom. Dry.—— | Damp. | | | Winds. | Atmos. Variation. |
|---|---|---|---|---|---|---|---|---|---|---|---|---|
| | 26 | 42 | 48 | 47 | $29^8$ | — | | 4 | 6 | 9 | NE.. | Fog .... R .. |
| | 27 | 48 | 50 | 47 | — | $30^2$ | | 11 | 10 | 8 | NE. | Fog ... |
| | 28 | 46 | 47 | 44 | — | $29^9$ | | 7 | — | 10 | SE.. | C ... |
| | 29 | 45 | 50 | 48 | | | | 7 | 9 | 10 | S. | C .. |
| | 30 | 43 | 53 | 51 | | | | 11 | — | — | SW.. | C .. |
| | 1 | 50 | 51 | 50 | $29^9$ | $^8$ | | 10 | 8 | 10 | W. | R .. |
| | 2 | 47 | 48 | 46 | 30 | — | | 10 | — | — | NW.. | C ... |
| ● | 3 | 49 | 49 | 48 | — | $30^1$ | | 12 | 10 | 8 | E. | Fog .... R ... |
| | 4 | 49 | 50 | 57 | — | 30 | | 9 | — | — | SE. | C ... |
| | 5 | 44 | 42 | 41 | — | $30^1$ | | 3 | 5 | — | SE. | F . |
| | 6 | 43 | 46 | 37 | $30^2$ | $^4$ | | 10 | 9 | 5 | NW.. | F .. |
| | 7 | 34 | 34 | 32 | $30^5$ | — | 1 — — | — | 6 | 5 | SE. | F .. |
| | 8 | 31 | — | 39 | $30^4$ | $^1$ | | 1 | 4 | — | N. | C .. |
| | 9 | 26 | 34 | 34 | $29^9$ | — | | 1 | 2 | 5 | N. | C .. |
| | 10 | 33 | 35 | — | $29^8$ | — | | 5 | 8 | 7 | NE | C . |
| ◐ | 11 | 35 | 37 | 33 | $29^9$ | 30 | | 1 | 3 | 6 | NE. | F .. |
| | 12 | 32 | — | 30 | $29^8$ | — | | 5 | 7 | 5 | NE.. | F . |
| | 13 | 28 | 30 | 28 | $29^7$ | — | | 5 | 3 | 6 | N... | F .. |
| | 14 | 30 | 35 | 32 | | | | 2 | 5 | 6 | N.. | F . |
| | 15 | 32 | 38 | 32 | $29^6$ | $^4$ | | 4 | 6 | — | NE. | F . |
| | 16 | 30 | 32 | 33 | $29^5$ | 29 | | 4 | 9 | 7 | N... | Snow .. |
| | 17 | 32 | 34 | 34 | $29^2$ | — | | 7 | 4 | 8 | SE. | R . |
| ○ | 18 | 35 | 35 | 37 | $29^3$ | $^4$ | | 7 | 8 | — | N. | Snow .. |
| | 19 | 39 | 41 | 40 | $29^5$ | — | | 8 | 7 | 8 | N. | C ... |
| | 20 | 39 | 36 | 34 | $29^6$ | — | | 11 | 10 | 8 | NW. | C .. |
| | 21 | 34 | 37 | 36 | $29^6$ | $29^8$ | | 7 | 10 | 9 | SW.. | C .. |
| | 22 | 39 | 38 | 35 | $29^9$ | — | | 6 | 9 | 7 | W. | C .. |
| | 23 | 37 | 30 | 37 | 30 | $30^2$ | | 6 | 7 | 7 | W.. | C .. |
| | 24 | 36 | 40 | 36 | $30^4$ | — | | 7 | — | — | NE. | C .. |
| ◑ | 25 | 35 | 36 | 32 | $30^4$ | — | | 2 | — | 7 | NE. | C ... S . C .. |

Quantity of rain from November the 25th to December the 25th, $\frac{78}{100}$. It will, probably, be considered that this interval has been remarkably dry, and on recurring to our register of last year, for the same period, we find that one inch $\frac{85}{100}$ of rain fell. This interval has also been characterised by an unusual continuance of frost, which lasted from the 6th to the 17th of December, the thermometer sometimes being as low as 24° in exposed situations.

The case of small-pox after vaccination mentioned in our last, and which is referred to by a correspondent as likely to be injurious, from being too indefinitely related, occured to a young lady at Hackney, and under the care, as we are informed, of Mr. Leese, who also vaccinated her. We hope the case will be fully explained by that gentleman; and then we have no doubt but it will be fully seen that it was unexpectedly mild, the susceptibility to the action of variola having been lowered by the previous vaccination.—Several cases of croup have occurred, and the various forms of pulmonic affections are becoming frequent.

MONTHLY

